# From concept to practice: real-world safety of JAK inhibitors in rheumatoid arthritis treatment

**DOI:** 10.3389/fmed.2026.1729135

**Published:** 2026-02-13

**Authors:** Karina R. Bonfiglioli

**Affiliations:** Rheumatology Division, Hospital das Clinicas da Faculdade de Medicina da Universidade de Sao Paulo, São Paulo, Brazil

**Keywords:** JAK inhibitors, JAK–STAT, Janus kinases, rheumatoid arthritis, targeted synthetic DMARDs

## Abstract

Janus kinase inhibitors have emerged as highly effective therapies, gaining a prominent role in rheumatoid arthritis treatment. However, by inhibiting multiple cytokine effector pathways, these agents have also raised concerns regarding their safety profile. This article aims to provide an updated overview of the main safety aspects of JAK inhibitors, with a particular focus on their use in RA.

## 1 Background

Over the last three decades, there has been significant progress in managing patients with Rheumatoid Arthritis (RA), particularly in early diagnosis, rigorous monitoring of disease activity, and target-oriented treatment strategies. A better understanding of pathophysiology has led to the development of new disease-modifying drugs (DMARDs), mainly biological DMARDs (bDMARDs), and more recently, targeted synthetic DMARDs (tsDMARDs). Therapeutic goals include controlling joint and systemic inflammation, slowing disease progression, preserving function and quality of life, and managing extra-articular manifestations, such as interstitial lung disease (1–4).

JAK inhibitors (JAKis), which are part of the tsDMARDs group, have demonstrated high efficacy in RA treatment. The currently approved agents for RA include tofacitinib (JAK1 and JAK3 inhibitor), baricitinib (JAK1 and JAK2 inhibitor), upadacitinib (selective JAK1 inhibitor), filgotinib (selective JAK1 inhibitor) (5) and peficitinib (JAK1, JAK2, JAK3 and Tyk2 inhibitor) (6).

JAKis target the JAK–STAT (Janus kinase - Signal Transducers and Activators of Transcription) signaling pathway, which transmits extracellular signals into the cell. Many cytokines rely on this pathway and can induce different responses depending on the cell type and the specific JAK units involved. The JAK family includes four tyrosine kinases: JAK1, JAK2, JAK3, and TYK2, which act in pairs to phosphorylate other intracellular proteins. JAKs bind to the intracellular portion of receptors as heterodimers and are activated when these receptors undergo conformational changes after binding their external ligand. Upon cytokine binding to its receptor on the cell surface, conformational changes trigger JAK phosphorylation and activation of the STAT system. This phosphorylation subsequently influences target genes, most of which are pro-inflammatory, leading to distinct intracellular responses (7–9).

**Figure 1:**
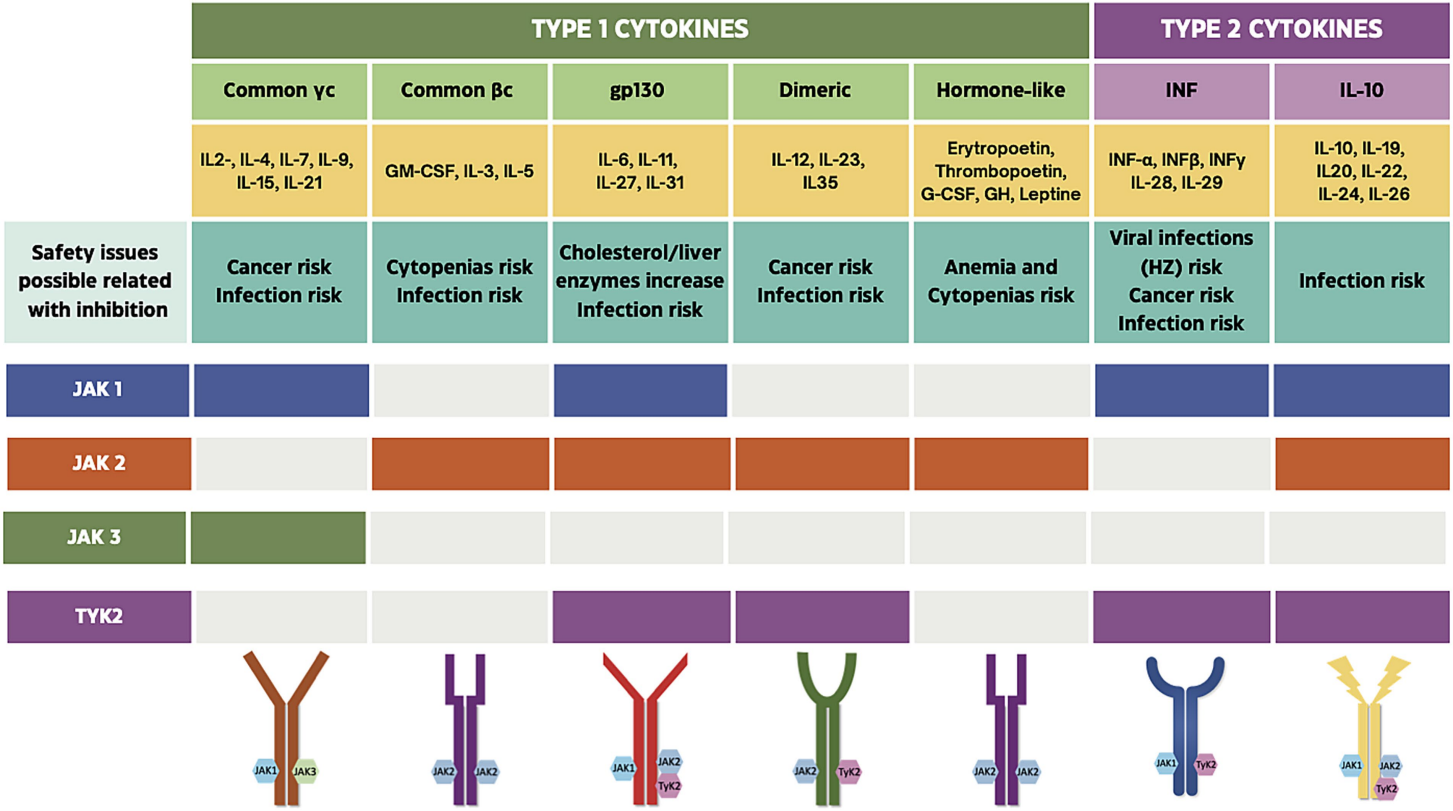
Overview of the JAK–STAT signaling pathway. The JAK–STAT system is activated by signal transduction through type I and type II cytokine receptors, initiating downstream phosphorylation cascades that regulate diverse biological processes including immune activation, inflammation, and hematopoiesis (7, 10).

JAK–STAT system is activated by signal transduction of type I and II cytokine receptors (7, 10). A detailed description, as well as their key biological functions, is provided below and summarized in Figure 1.

### 1.1 Type I cytokine pathways

- γc chain: Ligands IL-2 (enhances effector and regulatory responses), IL-4 (Th2 response), IL-7 (T-cell development), IL-9 (Th9 and mast cells, participates in atopic diseases) IL-15 (memory T cells and NK growth), IL-21 (differentiation of follicular helper T cells);
- βc chain: GM-CSF (macrophages and granulocytes proliferation, stimulates T-cell and dendritic- cell function), IL-3 (myeloid lineage proliferation, multipotent hematopoietic cells differentiation), IL-5 (Th2 response and eosinophilic regulator).
- gp130: Ligands IL-6 (pivotal in much of the inflammatory processes, involved in many autoimmune diseases), IL-11 (hematopoiesis), IL-27 (B-cell and T-cell regulation), IL-31 (Th2 response);
- Dimeric, or Th17 response pathway: Ligands IL-12 (Th1 response), IL-23 (Th17 response), IL-35 (anti-inflammatory responses);
- Hormone-like pathway: Ligands Erytropoetin (erythropoiesis), Thrombopoetin (differentiation of megakaryocytes and platelets), G-CSF (stem cells and granulocytes production in bone marrow), GH (cell division of condrocytes and IGF-1), Leptine (energy, homeostasis).

### 1.2 Type II cytokine pathways

- INF: Ligand IFNα, INFβ, IFNγ (amplifies infections defense, drives autoimmunity);
- IL10: Ligands IL-10 (anti-inflammatory responses), IL-19 (B-cell activation), IL-20 (synoviocyte migration, osteoclast development), IL-22 (amplifies IL-17 function), IL-24 (modulates T cells, B cells, NK cells, and macrophages), IL-26 (Th17 induced, bacteriostatic response).

JAK isoforms have distinct biological roles (Figure 1): In brief, JAK1 and TYK2 primarily mediate inflammatory signal transduction, whereas JAK2 and JAK3 are more closely involved in hematopoietic regulation. Accordingly, selective JAKi preferentially target a single isoform—most commonly JAK1—aiming to suppress inflammatory cytokine signaling, while limiting off-target effects (11). In contrast, non-selective JAKi simultaneously inhibit multiple JAK isoforms, resulting in broader cytokine blockade, but potentially related to side effects (11, 12). However, some considerations should be noted:

- As JAK enzymes work cooperatively, JAK1 can pair with any of the other three isoforms; therefore, even JAKis that are relatively JAK1-selective may have biological effects on all pairings involving JAK1 (11, 12).
- *In vitro* cytokine activity analysis of both healthy and RA individuals showed that kinase inhibition does not always translate to cellular JAK/STAT signaling, and most JAKi display predominant JAK1-mediated pathways (13).
- JAK isoform selectivity is dose and tissue dependent, and is thus relative rather than absolute (11).

Although most approved agents preferentially affect JAK1-dependent pathways, pharmacological data suggest that greater selectivity may translate into a more favorable safety profile, particularly regarding hematologic and immune-related adverse events, while differences in real-world safety are still being explored (11, 12).

## 2 Major safety concerns: bridging molecular mechanisms and real-world data

### 2.1 Serious infection

Given the broad spectrum of cytokine effector pathways targeted by JAKi, the potential risk of serious infections (SI), defined as those requiring hospitalization or intravenous antibiotics, has been a major safety concern. While accumulating data suggest that overall infection rates are generally comparable to those of other targeted therapies and largely influenced by patient-related factors, substantial heterogeneity among study populations limits the strength of direct comparisons. Aside from age, ohter subject aspects have been identifyed as risk factors for SI: underlying disease (with RA presenting higher SI rates), glucocorticoid (GC) dose, geographic region, male sex, body mass index and lymphopenia (14–16). Baseline risk assessment appears to influence outcomes more strongly than the choice of therapeutic class, particularly in patients with rheumatoid arthritis, for whom the disease itself confers an inherently higher risk.

*Findings from Randomized controlled trials (RCT):* In a *post hoc* analysis of the integrated upadacitinib clinical trial program encompassing approximately 27,000 patient-years, among patients with RA, rates of serious infections were comparable between upadacitinib (2.9 per 100 patient-years) and adalimumab (2.4 per 100 patient-years), and were numerically lower in the methotrexate group (1.3 per 100 patient-years) (17).

In pooled clinical trial and long-term extension data across rheumatoid arthritis, atopic dermatitis, and alopecia areata, baricitinib was associated with low rates of serious infections in low-risk patients with rheumatoid arthritis, defined as those younger than 65 years without predefined risk factors (1.73 per 100 patient-years), whereas at-risk patients, defined by age 65 years or older and or the presence of at least one cardiovascular or metabolic risk factor, showed higher but still moderate rates (2.95 per 100 patient-years), highlighting the influence of baseline risk on serious infection incidence (18).

In the ORAL Surveillance study, which specifically enrolled patients with rheumatoid arthritis aged ≥ 50 years, dose-dependent increases in the incidence of all infections were observed with tofacitinib compared with TNF inhibitors. Crude hazard ratios (cHRs) for tofacitinib compared with TNF inhibitors, in patients aged ≥50 and ≥65 years, respectively, were 1.29 (95% CI, 0.94–1.79) and 1.08 (95% CI, 0.74–1.58) for the 5 mg twice-daily dose, and 1.44 (95% CI, 1.05–1.99) and 1.55 (95% CI, 1.10–2.19) for the 10 mg twice-daily dose. The incidence rates (IRs) per 100 patient-years were 2.9, 3.6, and 2.4 in the overall trial population, and 4.0, 5.9, and 3.7 among patients aged ≥65 years, for tofacitinib 5 mg twice daily, tofacitinib 10 mg twice daily, and TNF inhibitors, respectively. Across treatment arms, the most consistent predictors of serious infections included older age, baseline opioid use, chronic lung disease and concomitant corticosteroid therapy, whereas non-serious infections were more common in women, past smokers, and those with higher disease activity or prior respiratory comorbidities. It is important to note that this trial was conducted in a population already at increased baseline risk for infection, which limits the applicability of its findings to the broader RA population (19).

*Findings from Real world evidence (RWE), Registers and Observational studies:* JAKi are associated with a modestly increased risk of overall infections and SI compared to TNFi and other bDMARDs in patients with rheumatoid arthritis (20, 21). A common denominator across most studies is the presence of baseline risk factors, including diabetes, age ≥65 years, prednisone use >5 mg/day, and also interstitial lung disease (22). However, this risk appears to vary according to the characteristics of the study design and population, ranging from no difference compared with TNFi or other bDMARDs (23–25) to more than a threefold increased risk (HR 3.45) in direct comparisons with interleukin-6 inhibitors (22). In a large real-world database of 130,718 patients with rheumatoid arthritis and over 100,000 person-years of follow-up, hospitalization for SI was the primary outcome, adjusted for confounders. HR for SI associated with tofacitinib was 1·41 (95% CI 1·15–1·73) vs. etanercept, 1·20 (0·97–1·49) vs. abatacept, 1·23 (0·94–1·62) vs. golimumab and 1·17 (0·89–1·53) vs. tocilizumab. The SI risk with tofacitinib was similar to adalimumab (1·06, 0·87–1·30) and certolizumab (1·02, 0·80–1·29), and was lower than infliximab (0·81, 0·65–1·00) (26).

*Findings from Systematic literature review (SLR):* A recent SLR on JAKi safety outcomes found no significant increased risk of SI in several comparative analyses with bDMARDs or csDMARDs, including clinical trials, safety trials, registries and observational cohorts (15). Additionally, a SLR focused on safety in comparison with TNFi showed that rates of SI were similar between JAKi and TNFi, with pooled hazard ratios close to 1.05, whereas the incidence of non-serious infections and herpes zoster was consistently higher with JAKi (27).

### 2.2 Herpes zoster

JAKis increase the risk of herpes zoster by suppressing key antiviral T cell–mediated pathways. They inhibit JAK-dependent cytokine signaling, particularly type I interferons (IFN-*α* and IFN-*β*), which drive innate antiviral defenses, and type II interferon (IFN-*γ*), crucial for Th1 and cytotoxic adaptive responses against varicella-zoster virus (VZV). JAK inhibition reduces CD4 + and CD8 + T cells producing IFN-γ, downregulates transcription factors such as STAT1 and T-bet, and increases regulatory T cells (Tregs), collectively impairing immunosurveillance and facilitating VZV reactivation (28).

Herpes zoster (HZ) reactivation is the most common infectious complication with JAKi Incidence rates range from 3–4 (Western Europe, US, Australia) to 9 (Japan, Korea) per 100 patient-years, compared to 2–3 per 100 patient-years for anti-TNF agents. Risk factors, other than East Asian ethnicity, include age, female sex, prednisolone/prednisone doses above 7.5 mg/day, recent infections, and hospitalizations. Severe events tend to be associated with higher doses of JAKis (29).

*Findings from RCT:* Clinical trials had already indicated an increased risk of HZ across the class, regardless of the degree of JAK selectivity, and in a dose-dependent matter. Data from the SELECT studies and integrated safety analyses consistently demonstrate that the use of upadacitinib is associated with an increased risk of HZ infection compared with placebo and other immunosuppressive agents (17). In rheumatoid arthritis, the incidence of HZ was higher in the groups receiving upadacitinib (both 15 mg and 30 mg) compared with methotrexate and adalimumab, showing a clear dose-dependent effect. Most HZ cases in the upadacitinib 15 mg and 30 mg groups were non-serious (96 and 93%, respectively) and limited to a single dermatome (74 and 76%) (30). In the ORAL Surveillance study, which already enrolled a population at higher baseline risk, the cumulative probability of HZ increased mainly during the first 6 months of treatment and then plateaued, suggesting an early phase of heightened susceptibility followed by stabilization over time (19).

*Findings from RWE:* Findings from observational studies are consistent with those from clinical trials. Reported relative risks varied across cohorts, with weighted hazard ratios ranging from 3.66 (95% CI, 2.38–5.63) for JAKi vs. csDMARDs in the German RABBIT registry (31), to 4.00 (95% CI, 1.59–10.06) for tofacitinib vs. etanercept in the Swedish multicenter RA cohort (32). In a retrospective cohort of RA patients treated with JAKi, the incidence of HZ was 5.11 per 100 patient-years, with higher risk associated with longer treatment exposure, prior HZ or COVID-19, and concomitant high-dose corticosteroid use. Earlier onset was observed in patients with a previous history of HZ (33).

*Findings from SLR:* A recent systematic review evaluating safety outcomes corroborated an increased incidence of HZ among patients treated with JAKi, as consistently observed across randomized controlled trials and observational registries (15). Although current evidence suggests a consistent class effect, a network meta-analysis of 47 randomized controlled trials involving 24,142 patients with immune-mediated inflammatory diseases found a risk more pronounced with certain JAKi—particularly peficitinib, baricitinib, and higher-dose upadacitinib—most notably in RA patients (34).

*Vaccination:* Considering preventive measures, recent studies have evaluated the immunogenicity and effectiveness of the recombinant zoster vaccine (Shingrix®, RZV) in patients treated with JAKi. In rheumatoid arthritis (RA) cohorts, humoral and cellular immune responses were generally preserved, though somewhat reduced compared with healthy controls. In a Swedish study including 82 RA patients on JAKi, approximately 80% achieved a ≥ 4-fold increase in anti-gE antibody titers after two doses, compared to about 98% in healthy individuals, with lower responses observed in those receiving concomitant methotrexate (35). Similar findings were reported in a sub study of SELECT-COMPARE, where 87.8% of patients on upadacitinib plus methotrexate mounted an adequate humoral response at week 16, and 71.4% maintained it at week 60 (36). Real-world data from a large inflammatory arthritis cohort also demonstrated reduced incidence rates of HZ among vaccinated patients (7.4 vs. 14.8 cases per 1,000 person-years), supporting vaccine effectiveness, albeit at lower levels than in immunocompetent populations (37). Overall, while JAKi may attenuate vaccine responses, RZV remains immunogenic and clinically beneficial, particularly in high-risk RA patients.

### 2.3 Malignancies and non- melanoma skin cancer

The rationale for concern regarding malignancy risk with JAKi stems from their impact on immune effector functions critical for tumor surveillance and cellular growth control. *In vitro* models demonstrate that the JAK/STAT pathway coordinates intercellular communication between tumor cells and the immune microenvironment, and its inhibition leads to reduced expression of proteins involved in cell proliferation, survival, and evasion of immunosurveillance mechanisms. Such interference may compromise cytotoxic T-cell and natural killer cell activity, potentially increasing oncogenic risk, particularly in individuals with predisposing factors (advanced age, prior immunosuppression, or high disease activity) (29, 38). The increased risk of non-melanoma skin cancer (NMSC) is particularly associated with inhibition of interferon and interleukin signaling pathways, which are critical for the detection and elimination of dysplastic or pre-malignant keratinocytes. This immunosuppressive effect is especially relevant in patients with prior ultraviolet (UV) exposure or other risk factors for cutaneous malignancy, as it may facilitate the development or progression of NMSC. As with solid malignancies in general, the risk of NMSC appears to become more consistent with longer treatment duration, and baseline factors, particularly older age, seem to play a key role in modulating susceptibility.

*Findings from RCT:* Clinical trials and observational data highlight that JAKi, especially tofacitinib and baricitinib, are associated with a modestly increased risk of malignancy compared to TNFi and other biologic DMARDs, with the risk most pronounced for lung cancer and NMSC, and in patients with prolonged exposure or multiple prior DMARDs (14, 16, 18, 39, 40). The ORAL Surveillance trial identified a higher incidence of lung cancer with tofacitinib (cHR 2.17; 95% CI 0.95–4.93), representing the most frequently observed malignancy in the tofacitinib arm, whereas breast cancer predominated among TNF inhibitor users (16). Regarding NMSC, increased risk were observed in the ORAL Surveillance trial, and the incidence rates were comparable between the safety trial and RCT programs (16, 37). The most frequently reported NMSC types were basal and squamous cell carcinomas, with unexpectedly high rates of squamous cell carcinoma in particular (16).

*Findings from RWE:* The RABBIT cohort demonstrated a higher risk of malignancy with JAKi compared with bDMARDs, with an adjusted hazard ratio of 1.40 (95% CI 1.09–1.80). This increased risk was evident only in treatment episodes exceeding 16 months and appeared more pronounced in certain subgroups, particularly among patients aged ≥60 years, three or more prior conventional synthetic DMARD therapies and high disease activity (40). Similarly, the North American case–control SEER Medicare database study demonstrated that JAKi use was associated with a significantly increased risk of lung cancer (OR 1.40, 95% CI 1.06–1.87), particularly among males (OR 2.12, 95% CI 1.14–3.94) and in those with more than 2 years of exposure (OR 1.52, 95% CI 1.01–2.28). In contrast, a lower risk of breast cancer was observed among females receiving JAKi (OR 0.62, 95% CI 0.39–0.97).

Other observational studies have yielded conflicting findings: in the STAR-RA cohort, the weighted hazard ratio for tofacitinib vs. TNF inhibitors was 1.15 (95% CI 0.96–1.39) (41), with no significant risk differences in U. S. and Taiwanese claims-based analyses (42, 43). In contrast, a Swedish prospective cohort reported an adjusted HR of 1.39 (95% CI 1.01–1.91) for JAKi (predominantly tofacitinib and baricitinib) compared with TNF inhibitors, but not for non-TNFi biologics (adjHR 1.00; 95% CI 0.78–1.28), with a trend toward increasing risk over time during a median follow-up of approximately 2 years (44). Data from a South Korea’s nationwide claims database also found a HR of 1.61 (1.08–2.41) in 2013–2013 registries from a RA cohort that included 14.972 patients, with 4,759 initiating JAKi (23).

It is important to note that most studies reporting an increased risk of malignancy involved long-term follow-up, typically exceeding 18 months. Therefore, extended observational periods are required to better delineate the true magnitude and temporal pattern of cancer risk associated with JAKi therapy.

*Findings from SLR:* In a meta-analysis of 18 clinical trials including 111,260 patients with rheumatoid arthritis, JAKi were associated with a significantly increased risk of malignancies, including overall cancer (RR 1.53, 95% CI 1.18–2.00), malignancies excluding non-melanoma skin cancer (RR 1.30, 95% CI 1.05–1.60), and non-melanoma skin cancer (RR 1.54, 95% CI 1.23–1.93) (45).

### 2.4 Major cardiovascular events

Cardiovascular diseases are the leading cause of death in RA and are related both to aspects of the disease itself and to traditional risk factors (46). JAKi may influence cardiovascular risk through complex and mechanism-based effects on immune-mediated and vascular pathways. As discussed by Zavoriti (47), unlike biologic agents, JAKi have been associated with a higher incidence of adverse cardiovascular events in patients with rheumatoid arthritis at increased baseline risk. By targeting cytokines that signal directly through the JAK–STAT pathway, JAKi profoundly modulate inflammatory signaling; however, key pro-inflammatory mediators including TNF, IL-17 and IL-1β act independently of JAK-associated receptors. Importantly, these non–JAK-dependent cytokines may still indirectly amplify JAK–STAT signaling and exert significant vascular effects. In particular, the combined activity of TNF and IL-17 promotes endothelial dysfunction, upregulation of adhesion and coagulation molecules, and a strongly pro-thrombotic milieu, independently of JAK signaling. Experimental data further suggest that JAK inhibition does not prevent—and may even exacerbate at higher concentrations—the pro-coagulant effects induced by TNF and IL-17, including suppression of physiological anticoagulant pathways. Collectively, these mechanisms provide a biologically plausible explanation for the less favorable cardiovascular profile observed with JAKi compared with biologics (47). Another suggested mechanism evolves enhanced platelet activation and increased thromboxane A₂ production, further promoting platelet aggregation and vasoconstriction (48). Recent data also suggest that Janus kinase inhibitors (JAKis) may promote immunothrombosis, a process linking inflammation and thrombosis, rather than classical inherited coagulopathy. In rheumatoid arthritis (RA) leukocyte assays, JAKis amplified Toll-like receptor 4 (TLR4)-induced cytokine release and accelerated clot formation, particularly in patients with active disease (49).

*Findings from RCT:* Although pivotal clinical trials did not initially show differences in the occurrence of major adverse cardiovascular events (MACE), subsequent clinical and pharmacovigilance data have revealed increased rates of MACE among patients treated with JAKi, particularly those with pre-existing cardiovascular risk factors or advanced age. These post-marketing observations prompted boxed warnings from regulatory agencies and underscore the importance of thorough baseline cardiovascular risk assessment, individualized treatment selection, and ongoing monitoring throughout therapy (50, 51).

The ORAL Surveillance trial demonstrated a numerical increase in MACE (including cardiovascular death, nonfatal myocardial infarction, and nonfatal stroke) with tofacitinib compared to TNF inhibitors, and noninferiority to TNFi was not achieved (cHR 1.33; 95% CI, 0.91–1.94 for combined tofacitinib doses vs. TNFi). The study enrolled a large at-risk population of 4,362 rheumatoid arthritis patients, followed for over 3 years. Within this cohort, MACEs occurred predominantly in older patients with a history of smoking or pre-existing cardiovascular risk factors. The risk difference between JAKi and TNFi was most evident in high-risk subgroups, with a cHR of 1.98 (95% CI, 0.95–4.14) among patients with prior atherosclerotic cardiovascular disease (ASCVD), compared to 1.14 (95% CI, 0.73–1.78) in those without ASCVD (16).

Extension studies of clinical trials did not confirm these findings; however, it is important to note that their design was not intended to assess non-inferiority. In a large integrated long-term analysis of 3,770 patients with rheumatoid arthritis, baricitinib was associated with low and stable rates of major adverse cardiovascular events, including in patients aged ≥50 years with cardiovascular risk factors, with no dose-related differences or new cardiovascular safety signals identified over up to 9.3 years of follow-up (18). An integrated analysis of clinical trials of upadacitinib, comprising approximately 27,000 patient-years across multiple rheumatologic indications, also did not identify an increased cardiovascular risk (17). Data from clinical trials of filgotinib and peficitinib also did not demonstrate an increased risk of MACE or overall cardiovascular risk (6, 52).

*Findings from RWE:* several recent real-world studies, including large cohort and claims-based analyses, have further explored cardiovascular outcomes associated with JAK inhibitor use. Notably, the STAR-RA study (53), together with findings from the US Corrona registry (43) and prospective multicenter cohorts from Sweden (32), Germany (54) and Italy (55, 56) have provided complementary evidence from routine clinical practice, offering valuable insights into cardiovascular safety beyond controlled trial settings. Overall, these analyses showed no evidence of an increased cardiovascular risk with JAKi compared to TNF inhibitors or other biologic agents, after adjustment for conventional cardiovascular risk factors and disease-related variables.

The STAR-RA study performed a subanalysis similar to the ORAL Surveillance trial, simulating the clinical trial setting in a high-risk population. In the real-world evidence cohort (*n* = 102,263; 12.6% tofacitinib initiators), the pooled weighted hazard ratio (HR) for tofacitinib vs. TNF inhibitors was 1.01 (95% CI 0.83–1.23), while in the randomized controlled trial–duplicate cohort the HR was 1.24 (95% CI 0.90–1.69). These estimates closely aligned with the ORAL Surveillance results (HR 1.33, 95% CI 0.91–1.94), indicating a numerically higher—but statistically non-significant—risk of major adverse cardiovascular events. Importantly, unlike the ORAL-Surveillance trial, in which a pre-specified non-inferiority margin with an upper 95% confidence interval threshold of 1.8 was defined, such an interception was not applied in the STAR-RA study. This methodological difference may partly explain the divergent conclusions between studies, particularly beyond the observation of a non–statistically significant increase in cardiovascular risk.

*Findings from SRL:* A recent meta-analysis reviewing studies published since 2019 on JAKi safety outcomes demonstrated that cardiovascular risk appears to be influenced also by dose in certain scenarios (including the underlying disease and the specific JAK inhibitor used), as well as by baseline risk factors, particularly advanced age (15). Another SRL evaluating 14 RCTs including 13,524 patients with rheumatoid arthritis found no increased risk of major adverse cardiovascular events with JAKi compared with placebo or other intervention, but identified a higher all-cause mortality risk vs. adalimumab, driven primarily by tofacitinib (57). Another recent meta-analysis did not demonstrate an increased risk of cardiovascular events, although it did identify an increased risk of malignancies (45).

### 2.5 Venous thromboembolism

Thromboembolic risk is generally elevated in patients with autoimmune diseases and is further influenced by baseline cardiovascular risk, age, prior venous thromboembolism, and concomitant therapies such as glucocorticoids and oral contraceptives (29). Similarly to MACE, a higher VTE risk related to JAKi may be related to mechanism-related effects on immune and vascular pathways. Recent mechanistic evidence suggests that JAKi (particularly those targeting JAK1 and TYK2) may amplify immune cell–mediated thrombosis in rheumatoid arthritis. Exposure to JAKi has been linked to increased tissue factor expression, NF-κB activation, elevated proinflammatory cytokines (TNF-*α*, IL-1β, IL17), and reduced levels of anticoagulant proteins such as protein S, collectively promoting a prothrombotic, hypercoagulable state, especially in patients with active disease (49). In addition, JAK inhibition has been associated with enhanced platelet activation and increased thromboxane A₂ (TXA₂) synthesis, a key driver of platelet aggregation and thrombosis, observed across different agents such as tofacitinib, baricitinib, and upadacitinib, and further amplified under systemic inflammation (48).

*Findings from RCT:* Interim analyses of the ORAL Surveillance trial (16) revealed increased rates of pulmonary embolism and all-cause mortality with tofacitinib 10 mg twice daily, findings not previously observed in efficacy trials, which prompted a mandatory dose reduction to 5 mg twice daily in 2019. A dose-dependent effect has been observed, although overall event rates in RCTs have been low, limiting conclusions regarding dose dependence (58, 59). Interestingly, in ORAL Surveillance, excess risk was primarily driven by pulmonary embolism (PE) rather than deep vein thrombosis (DVT) (16). On the other hand, long-term *post hoc* analyses of upadacitinib and baricitinib did not show an increased risk of venous thromboembolism. (17, 18), similarly to filgotinib and peficitinib (6, 52).

*Findings from RWE:* Pooled analyses of 14 registry and claims databases from Europe, the United States, and Japan also demonstrated a modest increase in VTE risk with baricitinib compared with TNF inhibitors (IRR 1.51; 95% CI 1.10–2.08), primarily driven by data from Swedish and French national cohorts (60, 61). The ARTIS registry reported adjusted hazard ratios (aHRs) of 1.73 (95% CI 1.24–2.42) for VTE, 3.21 (95% CI 2.11–4.88) for PE, and 0.83 (95% CI 0.47–1.45) for DVT in JAK inhibitor vs. TNF inhibitor users (61). In a case–control analysis within the French SNDS registry, JAK inhibitor exposure was associated with an increased risk of VTE both during treatment and up to 30 days post-discontinuation (IRR 8.27; 95% CI 3.41–20.04) (62).

*Findings from SRL:* Meta-analyses of randomized controlled trials and real-world cohort studies show that the absolute incidence of venous thromboembolism (VTE), including deep vein thrombosis and pulmonary embolism, in RA patients treated with JAKi is low—typically 3–8 events per 1,000 patient-years—comparable to or slightly above the background risk in RA populations (63, 64).

### 2.6 Laboratory abnormalities

The rationale for laboratory abnormalities observed in patients treated with JAKi is directly related to the broad inhibition of cytokine signaling pathways that regulate hematopoiesis, lipid metabolism, and immune cell function. JAK2 is essential for erythropoietin and thrombopoietin signaling, so its inhibition can lead to anemia and thrombocytopenia. JAK1 and JAK3 are involved in interleukin-15 signaling, which is critical for natural killer cell and lymphocyte proliferation; thus, JAK inhibition can cause lymphopenia and neutropenia. These cytopenias are typically mild to moderate but may be more pronounced at higher doses or with non-selective drugs. JAKi also alter lipid metabolism by interfering with cytokines such as IL-6, that suppress hepatic lipid synthesis, resulting in increased LDL-C and HDL-C levels. Liver enzyme elevations can also be observed, reflecting off-target effects on hepatic cytokine signaling (29, 65). Elevation of creatine phosphokinase (CPK) levels during JAKi therapy is a recognized phenomenon, though the underlying mechanisms are not fully understood. *Shojaiefard et al.* showed that JAK2 activity downregulates SLC6A8, reducing cellular creatine uptake, whereas JAK2 inhibition reverses this effect, increasing creatine entry into muscle cells and consequently raising plasma CPK levels (66). This mechanism suggests that JAK2 inhibition may enhance muscular creatine turnover or influx, resulting in asymptomatic laboratory CPK elevations rather than true muscle injury.

*Findings from RCT:* Most laboratory abnormalities with JAKi were mild to moderate and not associated with clinically meaningful adverse events. In contrast, parameters such as lipoproteins and transaminases exhibited consistent changes across all JAKi trials, supporting a class effect. Increases in LDL and HDL occurred in parallel, maintaining stable LDL: HDL ratios, without evidence of increased atherogenicity or CV risk, and remained responsive to statins (15). CPK elevations were relatively frequent, but usually mild and not linked to myositis or rhabdomyolysis (14, 15). Regarding hematologic parameters, slight decreases in hemoglobin were observed with upadacitinib and baricitinib (67, 68) while most other JAKi caused small increases (68, 69). Platelet counts transiently increased with baricitinib but generally decreased with other JAKi (68, 69). Changes in neutrophil and lymphocyte counts were mostly mild and transient, without an association with serious infections (67, 68).

*Findings from SLR: A* recent meta-analysis evaluated laboratory outcomes, and divergent effects were observed among several compounds, suggesting variability in downstream signaling across selective JAK–STAT pathways. Among hematologic parameters, slight hemoglobin reductions occurred with upadacitinib and baricitinib, whereas other JAKi showed modest increases. Platelet counts transiently rose with baricitinib but tended to decline with other agents. Variations in neutrophils and lymphocytes were mild and reversible, with no apparent link to serious infections (15).

*Age and gender matters:* https://bmjopen.bmj.com/content/15/12/e082366.long.

## 3 Discussion/conclusion

In light of the above, a risk profile for most adverse events can be outlined, typically including patients over 65 years, with high baseline cardiovascular risk, chronic glucocorticoid use, low mobility, current or past smoking, and pre-existing risk conditions (including lung disease, hepatic or renal impairment, and a personal or family history of cancer). Although JAKi should be used with caution in this scenario, persistent disease activity may represent a greater risk than the therapy itself.

Real-world studies and post-hoc analyses of clinical trials consistently identify older age as an independent risk factor for treatment discontinuation and serious complications, particularly in the presence of additional cardiovascular risk factors. In contrast, patients <65 years without significant comorbidities generally experience lower rates of serious adverse events and better drug retention, supporting EULAR recommendations to preferentially use JAKi in this lower-risk population (70). Evidence to date shows no clinically meaningful differences in safety or effectiveness between male and female patients, and minor sex-related variations in composite disease activity indices do not impact overall outcomes or treatment continuation (71).

A comprehensive risk-mitigation strategy should be adopted to minimize potential adverse events associated with JAKi. Pretreatment screening is essential to identify comorbidities and latent infections that may increase susceptibility to complications. Clinicians should remain aware of current warnings and contraindications, tailoring therapeutic decisions to each patient’s individual risk profile. Close and continuous monitoring is required throughout treatment to allow early detection of adverse effects, while dose optimization may help balance efficacy and safety, particularly in patients with risk factors or during long-term therapy. Finally, recognizing class-related complications and ensuring prompt intervention when they arise are key elements to maintaining treatment safety and maximizing therapeutic benefit (72). A detailed description of each of these steps is provided in Table 1.

**Table 1:** Strategies to reduce risks associated with JAK inhibitors (JAKi; (72)).

Screening domain	Recommended assessments
1. Carry out pretreatment screening	Patient history and examination with a focus on warnings and contraindications (see item 2) Routine laboratory testing: full and differential blood counts, liver blood tests, renal function, lipid levels. Viral Screening testing: Hepatitis B virus (HBV surface antigen, surface antibody, core antibody, and with/without HBV DNA testing) and hepatitis C virus (HCV antibody, with HCV RNA testing if antibody positive), HIV. Tuberculosis screening (as per national recommendations). Vaccination (as per national recommendations) - ensure vaccination against herpes zoster.
2. Keep in mind the warnings and contraindications	Severe active (or recurrent) infections, including tuberculosis and opportunistic infections. Current (or history of) malignancies. Organ dysfunction such as advanced chronic liver disease and severe renal disease (creatinine clearance <30 mL/min). Pregnancy and lactation. History of arterial and venous thromboembolic events. Vaccination with live vaccines.
3. Monitor carefully	Regularly check blood counts, renal and liver function, lipid profiles. Keep track of any signs of infections or adverse events. Regular skin examination (for detection of skin cancer)
4. Consider dose optimization	Dose adjustments in patients with higher age, significant renal or hepatic impairment, risk of drug interactions. Dose reduction in patients in sustained remission
5. Recognize class-related complications and implement prompt intervention	Serious infections, tuberculosis, herpes zoster (risk can be lowered with reduction/elimination of concomitant glucocorticoid use). Malignancy: rates may be higher with JAK inhibition compared with TNF inhibitors; the risk of non − melanoma skin cancer is elevated. Screening for cancer according to age group, gender, and risk factors is recommended. Lymphopenia, thrombocytopenia, neutropenia, and anemia may occur (this last one, particularly with JAK2 inhibition). Venous thromboembolic events (dose-dependent risk, especially pulmonary embolism, particularly when traditional risk factors are present) Elevations of creatine phosphokinase: usually not associated with clinical events. Elevations of creatinine: usually not associated with renal failure / hypertension.

The safety profile of JAKi is continuously evolving, alongside emerging indications for this highly effective and promising group in the management of autoimmune diseases. Understanding the complex networks underlying their mechanisms of action allows for the integration of real-world data and the anticipation of potential complications. Certain toxicities are now well established, enabling risk stratification according to individual patient profiles, particularly in rheumatoid arthritis, where baseline risk is inherently elevated.
